# Supplementary material for: Are carbon emissions trading and green financial instruments synergistic? -Comprehensive quantitative research based on content analysis
Source: PLoS One. 2024 Mar 7;19(3):e0298601. doi: 10.1371/journal.pone.0298601 (PMC10919601; doi:10.1371/journal.pone.0298601)
Supplement: S1 Data — (ZIP) [file pone.0298601.s001.zip › Data/地级市Z_F工作报告.zip!/╒π╜¡/╝╬╨╦╩╨/└┤╫╘╣1⁄2╓┌║┼í╛┬φ┐╦╩2╛▌═°í┐_2015.docx]

各位代表：

现在，我代表市人民政府向大会作工作报告，请予审议，并请市政协委员和其他列席人员提出意见。

2014年工作回顾

过去一年，市政府认真贯彻党的十八大和十八届三中、四中全会精神，深入实施省委“八八战略”和“干好一三五、实现四翻番”的决策部署，在中共嘉兴市委的正确领导下，认真执行市七届人大四次会议决议，坚持稳中求进工作总基调，着力抓改革、稳增长、促转型、治环境、惠民生，新常态下不断涌现新亮点。全市地区生产总值3352.8亿元，增长7.5%；财政总收入568.1亿元，其中一般公共预算收入307.1亿元，分别增长9.8%和8.8%；城镇居民和农村居民人均可支配收入42143元、24676元，分别增长9%和10.2%。

（一）致力稳增促调，经济保持平稳健康发展。实施扩大有效投资“三抓三保”专项行动，举办首届浙（禾）商大会，引进世界500强、国际行业领先企业和总投资超亿美元项目32个，实际利用外资24.96亿美元，浙商回归到位资金264.5亿元，完成固定资产投资2221.2亿元、增长16.3%。强化项目要素保障，消化利用供而未用土地1.9万亩。加强项目管理，完成182个市级政府投资项目竣工验收。深入推进工业强市建设，大力实施“四换三名”工程，完成企业技改投入662.3亿元，腾退低效用地1.2万亩，新增上市公司5家，规模以上战略性新兴产业、高新技术产业和装备制造业增加值分别增长12.8%、11.7%和15.7%。建筑业产值达到1134亿元。国家能源局在全国推广我市分布式光伏发电经验。平台功能得到提升，平湖独山港区和嘉善姚庄工业园区获批省级经济开发区。统筹做好稳出口、促消费等工作，外贸出口236.51亿美元、增长10%，社会消费品零售总额增长12.5%，网络销售零售额达到678.9亿元。服务业发展保持良好态势，接待国内外游客5391万人次，金融机构本外币存贷款余额突破10000亿元，服务业增加值占生产总值比重提高到41.6%。成功承办首届世界互联网大会。大力发展现代农业，新建粮食生产功能区15.1万亩、新增省级现代农业园区19个，粮食生产连续12 年保持稳定增长。国家创新型城市建设步伐加快，新建创新载体40个、院士专家工作站10家，引进国家和省“千人计划”人才37名，新增专利授权量1.7万件，全社会研究与试验发展经费支出占生产总值比重提高到2.5%。全市单位生产总值能耗及化学需氧量、二氧化硫、氨氮和氮氧化物等节能减排指标完成省下达目标任务，提前一年实现“十二五”淘汰落后产能目标。

（二）坚持先行先试，重点领域改革取得突破。深化行政审批层级一体化改革，428项省、市行政审批事项下放至县一级。海宁、嘉善在全省率先开展核准目录外企业投资项目不再审批改革试点。实行政府权力清单和责任清单制度，市级部门行政权力从11552项减少到3875项，明确市级部门主要职责492项。全面推进商事登记制度改革，新增各类市场主体5.8万个。在全省率先启动综合行政执法改革试点，组建市、县、镇三级综合行政执法机构。推进市场监管体制改革，组建市场监管局。总结海宁要素市场化配置改革试点经验并在全市推广，各县（市、区）全部列入省要素市场化配置改革试点。深化统筹城乡综合配套改革，加快农村产权制度改革步伐，新增流转土地面积12.1万亩，统筹城乡发展水平列全省第一。嘉善县域科学发展综合配套改革、平湖产业结构调整机制创新试点、桐乡全国旅游综合改革试点、海盐农村综合改革示范试点等各项国家、省改革试点进展顺利。获批国家新型城镇化综合试点。加强区域合作交流，实施深化接轨上海三年行动计划，积极参与杭州都市圈和浙东经济合作区共建，援藏援疆援青和“山海协作”等对口支援工作进展顺利。

（三）大力实施“五水共治”，生态环境治理取得积极成效。加强水环境治理，加大“清三河”力度，全面完成垃圾河清理1066.2公里，整治黑河、臭河847.8公里。加快污水处理设施建设，新建污水管网594公里，新增污水达标入网企业4152家。扎实推进农村生活污水治理，新增受益农户10.2万户。强化农业面源污染治理，实现生猪养殖业减量“三年任务两年完成”目标，各县（市、区）死亡动物无害化集中处理中心全面投运。全市跨行政区域河流交接断面水质考核首次获得优秀。加强饮用水安全保障，饮用水水源地水质明显改善。市域外引水工作取得重大进展。统筹抓好防洪、排涝、节水等工作，整治标准化圩区33.9万亩，加固海塘6.8公里。深入实施大气污染整治，组织开展秸秆禁烧专项行动，淘汰高污染小锅炉小炉窑665台、黄标车2.5万辆，市区空气质量优良率70.3%。加强固体废弃物处理与综合利用，市本级废旧商品回收体系建设加快推进。大力实施“四边三化”行动，新增改造绿化面积4.6万亩，新建生态绿道108公里。加强生态示范创建，新增国家级生态镇（街道）5个。

（四）统筹城乡区域发展，城市功能进一步提升。启动国家“多规合一”试点，开展新一轮市域总体规划修编。完善市域基础设施，杭平申线航道改造、嘉兴应急备用水源等项目扎实推进，建成钱江通道北接线一期、湖嘉申线航道一期等工程，嘉兴军民合用机场改扩建项目前期工作进展顺利。认真落实促进市本级经济发展的18条意见，完成市本级规划管理体制调整，国际商务区、湘家荡区域、温泉新城等重点区块开发建设步伐加快。全市启动城市有机更新10097亩，中心城区子城广场、南湖湖滨等区块房屋征收、设计规划和招商工作有序推进。大力实施“三改一拆”，积极开展无违建县（市、区）、无违建镇（街道）创建，改造旧住宅区、旧厂区、城中村1722.8万平方米，拆除违法建筑2013.8万平方米。开展城市交通治堵，大力实施道路综合整治工程，全市新增专用和公共停车位7925个，开发利用地下空间145.9万平方米。优先发展公共交通，中心城市新建公交场站40个，优化公交线路51条，公交分担率进一步提高。市区顺利完成“三小车”整治。加快滨海港产城统筹发展试验区建设，嘉兴港口岸扩大开放获得国务院批复，货物吞吐量、集装箱吞吐量分别达到6880万吨和115.6万标箱。支持各副中心城市特色发展，区域发展协调性进一步增强。联动推进4个省级和13个市级小城市培育试点工作，中心镇综合承载功能得到提升。大力实施农村土地综合整治，改造集聚农户1.4万户，复垦土地1.1万亩。深入开展美丽乡村“四级联创”，新增市级美丽乡村先进镇15个。

（五）着力保障和改善民生，社会建设水平不断提高。各级财政用于民生支出255.2亿元，占一般公共预算支出的76.2%。深化统筹城乡就业，新增城镇就业10.7万人，城镇登记失业率2.9%。加快社会保障提标并轨步伐，企业退休人员基本养老金待遇标准平均提高10%，城乡居民最低生活保障标准首次统一并提高到每月588元。完善住房公积金政策，开工建设保障性住房5485套。健全养老服务体系，新增机构养老床位2128张，居家养老服务照料中心城市社区、农村社区覆盖率分别达到89.2%和75.2%。各类教育协调发展，新建改建幼儿园46所，新增义务教育标准化学校34所、省级职业教育实训基地3个，浙江大学国际联合学院（海宁国际校区）开工建设。深入开展全国文明城市创建，城市文明程度指数测评连续两年居全省第一。启动国家公共文化服务体系示范区建设，大运河嘉兴段6段河道和2个遗产点列入世界文化遗产名录，成功承办第十二届全国美展综合画种·动漫展区展览。深化公立医院改革和基层卫生综合改革，推进优质医疗资源“双下沉、两提升”，认真做好重大传染病疫情防控工作，实现国家卫生城市创建县（市、区）“满堂红”。完成省荣军医院迁建。加强“体育强市”建设，大力开展群众体育，嘉兴体育代表团在第十五届省运会上取得了144枚金牌的优异成绩。实施“单独两孩”政策，计划生育特殊家庭帮扶制度进一步完善。档案、史志、气象等工作不断加强，妇女儿童、青少年、残疾人、慈善等事业取得新成绩。完成全市第三次经济普查。被确定为全国首批社会工作服务示范地区。加强新居民管理服务，启动省居住证制度改革试点。健全安全生产责任体系，开展安全生产大排查大整治和食品药品安全整治专项行动，安全生产事故起数、死亡人数分别下降5.2%和9%。加强信访工作，完善矛盾纠纷调解机制，健全社会治安防控体系，荣获全省平安建设银鼎。深化双拥创建，首次实现县（市、区）省级双拥模范城（区）创建“满堂红”。国防动员、人防、民族、宗教工作得到加强，外事、侨务、港澳台等工作不断深化。

（六）扎实开展党的群众路线教育实践活动，政府自身建设取得新进展。坚决贯彻中央八项规定和省委“28条办法”、“六项禁令”，围绕反对形式主义、官僚主义、享乐主义和奢靡之风，扎实开展党的群众路线教育实践活动，政风建设成效明显。市政府发文减少24.7%，清理规范性文件480件，全市性大会减少27.1%，“三公”经费支出下降39.8%。推进政务公开，市、县两级政务服务网全面开通，公开各类政府信息6.2万条。扎实开展“双千双百”进村入企走访服务活动，一批影响基层、企业发展的问题得到解决。年初确定的10项民生实事项目顺利完成。严格遵守和执行各项法律法规，自觉接受人大法律监督、工作监督和政协民主监督，定期向市人大报告、向市政协通报工作，积极回应人大代表、政协委员对社会热点问题的关注，办理市人大代表建议321件、市政协提案379件。深入开展正风肃纪专项检查活动，机关效能建设得到加强。认真落实党风廉政建设责任制，强化行政监察和审计监督，严肃查处违纪违法案件，惩治和预防腐败体系建设深入推进。

各位代表，面对复杂多变的外部环境和转型升级的迫切要求，我们主动应对各种困难和风险挑战，较好地完成了市七届人大四次会议确定的主要目标任务，这一成绩来之不易，各级干部也是蛮拼的。这是省委、省政府和中共嘉兴市委坚强领导的结果，是全市上下凝心聚力、开拓奋进的结果。在此，我代表市人民政府，向全市人民、外来建设者，表示衷心的感谢!向人大代表和政协委员，向各民主党派、工商联、各人民团体、无党派人士、离退休老同志和社会各界人士，向驻嘉人民解放军和武警官兵，表示衷心的感谢！向关心支持嘉兴发展的香港和澳门特别行政区同胞、台湾同胞、海外侨胞和国际友人，表示衷心的感谢！

我们也清醒地看到，当前我市经济社会发展还存在不少问题，主要是经济下行压力加大，部分企业生产经营比较困难，投资后劲不足；结构调整困难较大，经济发展过多依赖低端产业、过多依赖资源要素消耗、过多依赖低小散企业的状况还没有根本改变；中心城市集聚辐射功能不强，城市综合竞争力有待提升；资源与环境约束日益趋紧，环境治理和保护的任务依然艰巨；城乡居民持续稳定增收机制有待完善，多元化群体利益的协调难度增大，社会和谐稳定依然面临不少压力。与此同时，一些政府工作人员的服务意识、服务能力有待加强，改进工作作风、密切联系群众的长效机制需要进一步完善。对此，我们要直面问题，采取有力措施，认真加以解决。

2015年目标任务和主要工作

今年是全面深化改革的关键之年、全面推进依法治国的开局之年，也是完成“十二五”规划、研究制定“十三五”规划的重要节点。根据市委七届九次全体（扩大）会议精神，今年政府工作的指导思想是：高举中国特色社会主义伟大旗帜，认真贯彻党的十八大和十八届三中、四中全会精神，深入学习贯彻习近平总书记系列重要讲话精神，全面落实省委“八八战略”和“干好一三五、实现四翻番”的决策部署，牢牢把握稳中求进的工作总基调，坚持以提高经济发展质量和效益为中心，主动适应经济发展新常态，坚定不移深化改革开放，坚定不移打好转型升级组合拳，坚定不移推进城乡区域统筹发展，切实保障和改善民生，保持社会和谐稳定，加快推进“三城一市”和“两美”嘉兴建设，努力完成“十二五”规划目标任务，为“十三五”发展奠定更加坚实的基础。

做好今年政府工作，一要主动适应新常态。全面把握经济发展新常态下速度变化、结构优化、动力转换等特征，切实把调结构转方式摆在更加突出的位置，找准规模与质量、速度与效益之间的平衡点，保持定力，积极作为，努力实现更高起点、更高标准、更高质量的发展。二要牢牢把握新机遇。抓住国家“一带一路”、长江经济带、上海自贸区建设等重大战略机遇，以及当前我市正在推进的各项国家、省改革试点机遇，不断集成改革红利、开放活力和创新驱动力，加快培育嘉兴新的发展优势。三要不断拓展新空间。充分发挥嘉兴综合比较优势，在推进新型城镇化、产业转型升级过程中，坚持调整存量和做优增量并举，积极培育经济新增长点，切实增强发展的后劲和可持续性。四要努力增进民生新福祉。始终坚持需求导向、问题导向和满意导向，着力解决群众最关心、最直接、最现实的民生问题，在更高水平上完善社会保障，发展社会事业，优化社会管理，努力满足人民群众对美好生活的新期待。五要扎实推进依法行政新实践。充分发挥法治引领和规范作用，坚持法定职责必须为、法无授权不可为，自觉运用法治思维和法治方式深化改革、推动发展、化解矛盾、维护稳定，不断提高依法行政的能力和水平。

建议2015年全市经济社会发展的主要预期目标为：地区生产总值增长7.5%左右；一般公共预算收入增长7.5%左右；城镇居民人均可支配收入增长8.5%，农村居民人均可支配收入增长9%；居民消费价格指数与省价格调控目标保持衔接；节能减排各项指标完成或超额完成省下达的目标任务。

重点工作是：

（一）突出提质增效，加快经济转型升级步伐

以扩大有效投入促转型。发挥投资对经济发展的关键作用，组织实施扩大有效投资“项目大推进、招商大引进、服务大跟进”专项行动，确保固定资产投资增长12%以上。优化投资结构，聚焦大项目、好项目带动，确保重大基础设施、重大产业、重大工业技改、重大生态建设和环境保护项目投资均增长20%以上。坚持国资、外资、民资“三资齐上”抓投入，注重发挥政府投资的导向作用，推出更多面向民间资本的优质项目。加强项目要素保障，继续做好供而未用土地消化利用工作，积极向上争取省重点项目土地点供指标。结合研究制定“十三五”经济社会发展规划，谋划储备一批符合我市长远发展需求的战略性项目。

以提升产业素质促转型。加快工业强市建设，重点围绕主导产业、特色优势产业等领域做强“十大产业链”，促进高端装备制造、新材料、新能源等产业形成更高水平的集群发展优势，力争规模以上战略性新兴产业产值达到3600亿元。深入推进“四换三名”工程，组织开展“两化”融合专项行动，实施“机器换人”项目900个，腾退低效用地1万亩，推动传统产业向中高端发展。扎实推进国家质量强市示范城市建设，抓好平湖、桐乡“浙江制造”试点工作。推进服务业提速提质发展，大力发展现代物流、科技服务、电子商务、健康养老等服务业，力争服务业增加值突破1500亿元，占生产总值比重达到42%。加快转变农业发展方式，积极发展多种形式的适度规模经营，支持种养大户、家庭农场和专业合作社发展，大力发展生态循环农业，加强农业科技创新和信息化建设，进一步提升农业产出效益。守住粮食安全红线，增强粮食保供能力。

以培育经济新增长点促转型。抓住乌镇被列为世界互联网大会永久会址的契机，加快发展以互联网产业为核心的信息经济，积极争创国家互联网产业创新发展综合试验区。大力发展时尚经济，充分运用创意设计、品牌运作等手段，推动服装、皮革、毛衫、箱包等传统优势产业向时尚化转型。大力发展海洋经济，深入推进港产城统筹发展，加快海河联运集疏运体系建设，培育壮大临港先进制造业集群，力争滨海新区规模以上工业产值达到1200亿元。大力发展军民融合产业。抢抓上海迪士尼开园机遇，加快旅游业转型发展，着力打造运河国际旅游休闲城市。按照企业主体、资源整合、项目组合、产业融合原则，加快规划建设一批特色小镇，以新理念、新机制、新载体推进产业集聚、产业创新和产业升级。适应消费结构升级趋势，大力发展网上交易、连锁经营、物流配送等商贸业态，积极培育消费热点。

（二）坚持改革引领，不断释放体制机制红利

深化要素市场化配置改革。建立完善公开公正的亩产效益综合评价机制，启动开展服务业企业绩效评价。推广要素分层定价、企业分类指导制度，积极推行土地、电力等资源要素差别化供给，全面开展城镇土地使用税政策调整工作。健全要素交易平台，深入推进土地、排污权、用能等节余指标交易，激励企业更好地盘活存量、优化增量。

深入推进统筹城乡综合配套改革。围绕“三权到人、权跟人走”，联动推进土地承包经营权、宅基地使用权和集体收益分配权改革，扎实推进农村产权确权登记颁证。健全农村产权交易市场体系，稳步推进农村产权市场化流转、抵押担保和入市交易。加强农村集体资产监管，全面完成村级集体经常性收入低于30万元的经济薄弱村转化工作。

加快建立现代财政制度。认真贯彻新《预算法》，健全预算管理制度，完善预算追加和调整管理办法，深入推进预决算公开。建立跨年度财政预算平衡机制，研究编制三年滚动财政规划。改革财政专项资金使用方式，加强绩效评估。规范政府性债务管理，切实防范和化解政府性债务风险。

深化投融资体制改革。推行负面清单管理模式，积极推广核准目录外企业投资项目不再审批等投资便利化措施。推广政府与社会资本合作模式，探索利用社会资本建设大项目的体制机制。加大政府购买公共服务力度，鼓励支持民办教育、民办医疗、民办养老等事业发展。加快金融创新步伐，积极推进多层次资本市场发展，支持地方法人金融机构做大做强，鼓励金融机构开展金融产品创新。

深化国资国企改革。优化国资布局，加快国有资产整合重组，加大主导产业和优势企业培育力度，鼓励和支持民营企业参与国有企业改革，推动国有企业转型发展。完善国资监管体系，健全监管制度和机制，逐步建立以管资本为主的监管方式。实施国资公司分类管理，加快建立以效益、净资产收益率和社会贡献率为主要指标的分类考核评价体系。

（三）强化创新驱动发展，加快建设创新型城市

加强创新平台建设。抓住嘉兴科技城被列入省级创新平台的机遇，加强创新资源整合，推进科技城扩容提质，着力打造科技与产业高度融合的创新核心区。支持平湖张江长三角科技城、秀洲光伏高新园区、嘉善归谷科技园、海盐中国核电城等平台建设，支持有条件的园区积极创建国家、省级高新技术产业开发区。深化省校合作模式，发挥好浙江清华长三角研究院、浙江中科院应用技术研究院等科研院所的作用，不断提升战略合作水平。完善专业化、社会化的科技公共服务体系，鼓励和引导社会资本参与“泛孵化器”建设，启动建设国家检验检测高技术服务业集聚区。

促进科技资本产业融合发展。加快省科技金融改革创新试验区建设，支持设立科技支行、科技小额贷款公司、科技保险公司等金融机构，扩大创投引导基金规模，不断健全科技投融资体制。突出企业创新主体地位，鼓励企业加大研发投入，建立研发机构，重点支持企业研究开发新产品、新技术。健全技术创新市场导向机制，加快建设科技大市场，推进市、县、镇科技成果转化管理服务体系建设。

集聚创业创新人才。深入实施“创新嘉兴·精英引领计划”，扎实推进人才改革试验区建设，引育国家、省“千人计划”人才35名以上。实施“人才强企”工程，注重企业家精神培育，加大高技能人才队伍建设力度。健全人才引进、培养、使用等工作机制，加快形成大众创业、万众创新的生动局面，努力把嘉兴打造成高层次人才创业创新的乐园。

（四）扩大对内对外开放，提升开放型经济发展水平

加大接轨上海力度。大力实施“与沪杭同城”战略，全力打造浙江省接轨上海示范区。做好与上海城市轨道交通的规划对接，加强嘉兴军民合用机场与上海机场集团合作，扎实做好沪乍杭铁路前期工作。提升临沪经济区开发建设水平，健全省级以上开发区、市镇工业园区与上海平台的长效合作机制。加快推进上海自贸区协作配套区建设，及时复制、借鉴上海自贸区的管理经验。深入实施一批合作办学、办医等社会民生项目，继续推进两地公交、医保等“一卡通”，努力实现与上海公共服务资源的互联共享。认真落实国家区域发展战略，继续做好援藏援疆援青等对口支援工作。

提高引资质量效益。深入实施招商选资和浙商回归“一号工程”，围绕重点产业、重点企业开展精准招商，引进世界500强、国际行业领先企业和总投资超亿美元项目20个，确保实际利用外资24亿美元以上，浙商回归到位资金288亿元。创新招商引资方式，整合招商资源，继续办好嘉洽会等主题招商活动，加强与境内外知名商会、投资促进机构合作，引导企业通过合资合作、产业链配套等方式引入投资者。加快各类开发区特色化、专业化、差异化发展，鼓励支持建设各类新经济园，启动中德工业园建设，力争嘉兴出口加工区获批国家综合保税区。

推动内外贸一体化发展。完善出口基地公共服务平台，协同推进货物贸易和服务贸易，推动跨境电子商务快速发展，重点支持高附加值产品、自主品牌产品出口。引导企业扩大先进技术装备、关键零部件和重要能源资源进口。进出口总额增长7%。加快“走出去”步伐，鼓励重点龙头企业到境内外建立营销网络，建立境外经贸合作区，支持企业开展国际并购和股权投资，大力培育跨国公司。鼓励外贸企业积极开拓国内市场，促进内外贸融合发展。

（五）加强生态文明建设，着力建设“美丽嘉兴”

深入推进治水治气。实施“消灭黑臭河、劣Ⅴ类水三年行动计划”，完成河道清淤1000公里，巩固垃圾河治理成果，力争全面消灭黑河、臭河。加快截污纳管进度，新建污水管网280公里，加快污水处理厂提标改造步伐，进一步加大工业企业污水全入网推进力度。加快农村生活污水治理设施建设，实现农村小集镇污水收集管网基本全覆盖。深化农业面源污染治理，完善生猪养殖业控量提质长效监管机制。全面落实“四位一体”保洁机制，严格水环境执法监管，巩固八大重点区域治理成果，促进水环境质量持续好转。扎实推进平湖塘延伸拓浚、扩大杭嘉湖南排等工程建设，继续实施圩区治理。加快市域外引水步伐，力争项目早日开工建设。加强大气污染整治，统筹推进工业废气、机动车尾气、秸秆焚烧、餐厨油烟、扬尘等“五气共治”，全面开展高污染锅炉专项整治，实施燃煤电厂清洁排放技术改造，推广秸秆多元化利用模式，全面完成黄标车淘汰任务。提高环境空气监测预警能力，完善大气污染应急响应体系。

大力实施节能减排。严格执行能源“双控”管理，严禁新上高耗能、高污染项目。深入开展工业循环经济示范园区创建，大力发展绿色环保产业，实施节能技术推广应用示范项目180项，完成80家企业清洁生产审核。深入做好餐厨废弃物资源化利用国家试点工作。调整能源结构，积极推广分布式光伏发电应用，继续做好“煤改气”、“油改气”等工作。加强重点企业排污总量监管，坚决打击超标准排污行为。

加强生态文明制度建设。以新《环保法》实施为契机，铁腕治污，依法严惩各类环境违法行为。严格执行主体功能区制度，加强生态环境空间管制，坚守生态保护红线。健全生态保护激励约束机制，开展区域资源有偿使用和生态补偿试点，推行主要污染物排放财政收费制度。完善排污权交易办法，实行排污总量削减替代制。创新环境治理方式，培育环境污染治理第三方市场。推进公众广泛参与的环保制度建设，及时公布环境信息，加强社会监督。

（六）扎实推进新型城镇化，促进城乡发展一体化

优化城市空间布局。开展国家新型城镇化综合试点，探索建立“多规合一”机制，修编完善市域总体规划、城市总体规划以及镇级总体规划，推动经济社会发展规划、城乡规划、土地利用规划和生态环境保护规划融合衔接，形成“一本规划，一张蓝图”。加强市域内重大功能区块、战略性资源统筹，促进主副中心城市一体化发展。加快县域城镇化进程，推动六个副中心做大规模、做强功能、做优环境，实现差异化、特色化发展。提升市域基础设施建设水平，加快推进杭州湾跨海大桥北接线二期、京杭运河三级航道整治等工程。

提升中心城市功能。坚持产城融合发展，实施城市组团开发，加快嘉兴科技城、国际商务区、湘家荡区域、温泉新城等重点区块建设。认真落实市本级产业发展规划，大力发展楼宇经济，积极引进和培育总部基地、新兴金融、文化创意等业态，推动县域经济向城市经济转型。高标准实施城市有机更新，加快中心城区子城广场、南湖湖滨、火车站等片区更新改造步伐，确保早出形象。继续推进“三改一拆”工作，深入开展无违建县（市、区）、无违建镇（街道）创建，重点抓好城郊结合部、工业园区等区域综合整治，带动重点区块改造提升。加大城市交通治堵力度，强化科技治堵、交通管理和社会参与，加快实施一批交通畅通项目，不断提高城市通行速度和群众满意度。积极争创省公交优先示范城市。加快建立“数字城管”应用体系，积极推行“路长制”，不断提高城市管理科学化、精细化、智能化水平。

加快建设美丽乡村。扎实做好省、市两级小城市培育试点工作，不断提升新市镇辐射带动能力。优化村庄规划布局，有序推进农村土地综合整治，完成土地整治复垦1.1万亩。加强农村建房管理和危旧房改造，积极开展省级农房改造示范村建设。深入实施美丽乡村“四级联创”，加强历史文化村落保护，积极创建一批优美庭院示范户。深入实施“四边三化”行动，大力开展平原绿化，新增改造绿化面积2.5万亩。

（七）繁荣发展各项社会事业，努力增进民生福祉

加强居民增收和民生实事工作。积极拓宽城乡居民增收渠道，千方百计提高城乡居民收入。完善促进就业、鼓励创业、自主择业的体制机制，建立就业困难人员实名管理服务制度，统筹做好重点群体就业工作，新增城镇就业6.5万人。实施高校毕业生创业引领计划，加快创业示范基地、创业园建设。完善企业工资集体协商、最低工资标准调整机制，促进职工工资稳步增长。健全为民办实事的长效机制，市政府今年将继续推出水环境治理、交通治堵、居家养老服务体系建设等10件民生实事项目。

健全社会保障体系。加快社会保险扩面提标，建立全民参保登记和动态管理机制，完善城乡居民基本养老和基本医疗保险制度，健全社会保障待遇确定和正常调整机制，实现城乡居民大病保险全覆盖。加快发展养老服务业，扎实推进城乡社区居家养老服务照料中心建设，积极推广医养结合的养老服务新模式。推进市老年公寓和残疾人康复中心建设。加强社会救助规范化建设，稳步提高对贫困家庭和困难人群的救助标准。保持房地产市场平稳健康发展，推进廉租房与公租房并轨运行，新开工保障性住房1960套。

提高教育事业发展质量。加快教育现代化步伐，深化教育领域综合改革，大力实施第二轮学前教育三年行动计划，推进义务教育标准化学校建设，利用信息化手段不断扩大优质教育覆盖面，促进基础教育高水平均衡发展。深入开展特殊教育提升计划，争创国家特殊教育改革试验区。加快职业教育体制改革，加大“五年一贯制”中高职统筹发展力度，推进职业教育产教融合、校企合作。支持嘉兴学院等在禾高校发展，加快推进浙江大学国际联合学院（海宁国际校区）建设。积极构建终身教育体系，努力建设学习型城市。

加快文化事业发展。弘扬社会主义核心价值观，高起点开展新一轮全国文明城市创建。推进基本公共文化服务标准化、均等化，加快城乡一体化文化馆总分馆服务体系建设，完善“文化有约”服务平台，力争成功创建国家公共文化服务体系示范区。做好大运河遗产保护工作，加强非物质文化遗产的保护、传承和利用。鼓励文艺精品创作，推出一批思想性、艺术性、观赏性有机统一的优秀作品。重视文化产业基地建设，积极培育文化创意、文化旅游、文博会展等业态，促进特色文化产业加快发展。

提升卫生体育等事业发展水平。深化公立医院改革，加强公共卫生服务资源整合，继续推进优质医疗资源下沉。提升社区卫生服务中心标准化建设水平，完善合理分级诊疗模式，不断提高基层就诊比例。广泛开展全民健身活动，加大公共体育设施向社会免费开放力度，加快发展体育产业，举办第八届市运会。加强人口计生工作，深入实施“幸福家庭关爱工程”，促进人口长期均衡发展。

（八）加强社会治理创新，深入推进“平安嘉兴”建设

发挥社会组织作用。加强新形势下群团工作，积极支持工会、共青团、妇联、科协、残联、红十字会等群团事业发展。创新社会组织管理体制，重点培育发展行业协会商会类、公益慈善类、科技类、社区服务类等社会组织，鼓励支持社会组织参与社会治理和公共服务。重视社会工作人才队伍建设，不断提升志愿服务专业化、社会化水平。

强化基层管理服务。健全社区服务体系，加强社区公共服务信息平台建设，完善城乡社区便民服务中心功能。全面实施居住证制度改革，强化新居民综合管理。引导和动员群众依法自我管理、自我服务，让村（居）委会集中精力开展村（居）民自治。

努力促进社会和谐。建立畅通的诉求表达、矛盾调处、权益保障机制，充分利用市长电话、政务微博等平台，不断拓宽社会公众民意表达渠道。加强和完善网络信息安全管理，营造良好的网络环境。加强“六五”普法教育，推进公共法律服务体系建设。开展社会矛盾大排查、大调解，有效化解社会矛盾。大力推行阳光信访，着力化解信访积案。

切实保障公共安全。健全隐患排查治理和安全预防控制体系，坚决遏制重大安全事故。完善食品药品安全监管方式，健全食品原产地可追溯制度。加强社会诚信体系建设，完善信用约束机制。健全突发事件预警和应急处置机制,提高应急处置能力。强化社会治安综合治理，依法严厉打击各类违法犯罪活动，着力打造长三角最具安全感的城市。大力支持驻嘉人民解放军和武警部队建设，推进国防后备力量和人民防空建设，力争实现全国双拥模范城市创建“三连冠”。

努力建设法治政府

党的十八届四中全会对建设法治政府提出了更高的要求，我们必须认真落实《中共嘉兴市委关于全面深化法治嘉兴建设推动法治建设走在前列的实施意见》，加快建设职能科学、权责法定、执法严明、公开公正、廉洁高效、守法诚信的法治政府。

一要加快职能转变。正确处理政府与市场的关系，最大限度地减少政府对微观事务的管理，加强政府公共服务、市场监管、社会管理、环境保护等职责，使市场在资源配置中起决定性作用和更好发挥政府作用。深化“四张清单一张网”改革，建立健全“四张清单”动态调整机制，重点推进权力清单“瘦身”、责任清单“强身”、政务服务网功能提升。深化行政审批层级一体化改革，进一步清理压缩审批事项，优化和规范审批流程，推动政府职能由注重事前审批管理向加强事中事后监管转变。推进政府机构改革，加强政府职能精细化管理，理顺权责关系。

二要严格依法行政。严格按照法定权限和程序行使权力、履行职责。健全行政决策机制，完善公众参与、专家论证、合法性审查、集体讨论决定等行政决策程序，建立决策后评估和纠错制度。健全政府法律顾问制度。深入推进综合行政执法改革，落实行政执法责任制，健全行政执法裁量权基准制度。全面推进政务公开，重点加强财政预决算、公共资源配置、重大建设项目批准和实施、社会公益事业建设等领域的政府信息公开工作。强化对行政权力的制约和监督，自觉接受人大监督、民主监督、司法监督，加强政府内部层级监督、专门监督和审计监督，主动接受社会监督、舆论监督。

三要切实改进作风。巩固扩大党的群众路线教育实践活动成果，高标准落实中央八项规定和省委“28条办法”、“六项禁令”，坚持不懈纠正“四风”。始终把人民意愿和关切作为政府工作着力点，进一步密切与群众的联系，增进与群众的感情，凝聚群众的智慧和力量，共同维护和促进来之不易的改革发展稳定的大好局面。完善深入基层调查研究制度，组织开展“转型发展服务年”活动，不断提高服务基层、服务企业、服务群众的针对性和精准度。大力倡导敢于负责、敢于担当、敢闯敢试的精神，加强对重大决策部署落实情况的督促检查，对确定的目标、部署的任务，一抓到底，确保政令畅通。从严管理公务员队伍，每一位政府工作人员要始终坚持谋利民之策、办亲民之事、解困民之忧，努力赢得人民群众的更多信任，获得人民群众的更大支持。

四要坚持廉洁从政。深刻领会习近平总书记“三严三实”要求，坚定理想信念，加强道德修养，坚持自重、自省、自警、自励，常修为政之德，常思贪欲之害，常怀律己之心。强化党风廉政建设主体责任，严格落实“一岗双责”。严格执行廉洁从政各项规定，厉行勤俭节约，反对铺张浪费，严格控制和审计公务消费。坚持用制度规范行政行为，形成靠制度管权管事管人的有效机制。强化对重点领域、关键环节权力运行的监督制约，对各类腐败行为保持“零容忍”，做到发现一起，查处一起，绝不姑息。

各位代表，新常态新征程已经开启，发展的号角催人奋进。大力推进“三城一市”和“两美”嘉兴建设，需要全体嘉兴人民的共同奋斗！只要我们的心往一处想，嘉兴的发展就有坚实的基础、坚强的动力和保障；只要我们的劲往一处使，嘉兴的发展就能适应新常态，迈向新征程，跨上新台阶；只要我们咬定青山不放松，就一定能肩负起时代赋予的光荣使命，创造光辉的业绩，实现人生的美好价值！各位代表，身处伟大的时代，面对伟大的梦想，让我们紧密团结在以习近平同志为总书记的党中央周围，在省委、省政府和中共嘉兴市委的坚强领导下，进一步解放思想，开拓创新，脚踏实地，攻坚克难，为谱写中国梦的嘉兴篇章而努力奋斗！
